# Pulmonary Sequestration Masquerading as Pulmonary Abscess

**DOI:** 10.7759/cureus.72579

**Published:** 2024-10-28

**Authors:** Baltej Singh, Ezekiel Kurcab, Kris Conde, Patricia E Simmer, Michael T Vest

**Affiliations:** 1 Internal Medicine, Christiana Care Health System, Newark, USA; 2 Radiology, Christiana Care Health System, Newark, USA; 3 Internal Medicine/Emergency Medicine, Christiana Care Health System, Newark, USA; 4 Internal Medicine/Critical Care, Sidney Kimmel Medical College, Philadelphia, USA; 5 Internal Medicine/Critical Care, Christiana Care Health System, Newark, USA

**Keywords:** broncho-pulmonary sequestration, computer tomography scan, congenital lung malformations, intralobar pulmonary sequestration, • lung abscess, ‘pulmonary infection’

## Abstract

Pulmonary sequestration is a rare congenital condition wherein a nonfunctional lung segment, arising separately from the true lung bud, develops within the chest cavity but lacks communication with the tracheobronchial tree or pulmonary arterial supply. While this condition is typically diagnosed in children, our case highlights its relevance in adults. We present a 37-year-old male who presented with shortness of breath and was initially diagnosed with a pulmonary abscess. A careful review of CT imaging, particularly noting the vascular supply to abnormal pulmonary lesions, led to the diagnosis of infected pulmonary sequestration. This abnormality had been noted previously, but the patient was unaware of the finding, leaving him at risk of infection and subsequent hospitalization. Although this can be a benign and incidental finding on imaging, this case emphasizes the need for appropriate diagnosis, patient education, and appropriate follow-up to avoid future complications.

## Introduction

Pulmonary sequestration is a rare congenital pulmonary condition that accounts for <6% of congenital lung malformations. Anatomically, it consists of a nonfunctional segment or lobe of lung tissue that does not communicate with the tracheobronchial tree and also does not share an arterial blood supply with the rest of the lung tissue. Worldwide prevalence has not been reported, but a population database from China 2010-2019 showed 4.2 cases per 100,000 live births with a slight preponderance for men vs. women at 4.5 vs. 3.8 per 100,000 [1]. Most patients are diagnosed at age 20 or younger. It is rarely ever diagnosed after age 50 [2].

Here, we present a case of an adult with pulmonary sequestration, initially diagnosed as a pulmonary abscess.

## Case presentation

A 37-year-old male long-haul truck driver with a history of diverticulitis status post-partial bowel resection in 2021 presented with the chief complaint of shortness of breath and dyspnea on exertion. His symptoms started about two days before presentation with a cough, which then progressed to shortness of breath and fatigue. This prompted him to interrupt his trip and present to the emergency department. 

He is a former cigarette smoker with an 11-pack-year history and quit vaping approximately three months before the presentation. He drinks alcohol very occasionally (one to two times per month) and denies any recreational drug use. He denies having any known history of asthma, chronic obstructive pulmonary disease (COPD), or any other lung disease. He has no family history of lung disease. 

On presentation, he was febrile to 38.8 C. His blood pressure was normal at 120/78 mm Hg, but he was tachycardic with a heart rate of 142 beats per minute, a respiratory rate of 22 breaths per minute, and a saturation of 95% on room air. His exam showed no distress but was notable for coarse breath sounds in the left lower lung fields. Laboratory data showed a normal white blood cell count (9100 per microliter), normal creatinine, and a positive polymerase chain reaction test for COVID-19.

**Figure 1:**
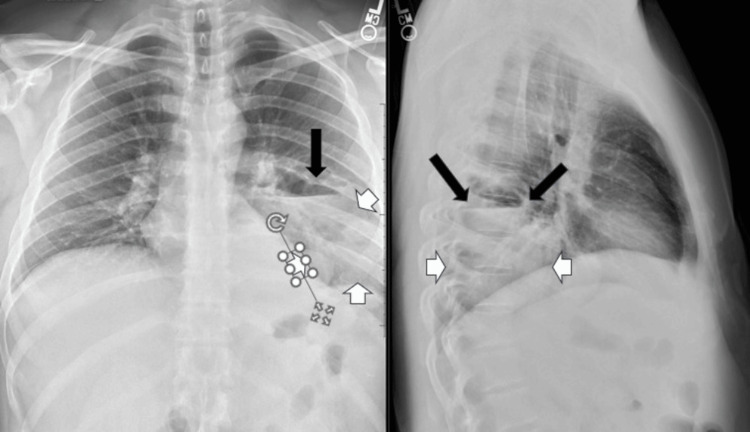
Admission chest X-ray This image shows posterior-anterior (PA) and lateral views of the admission chest X-ray. There is confluent opacity in the left lower lobe posterior thorax (short white arrows) with sharp air-fluid levels (long black arrows).

Figure 1 shows his initial chest X-ray revealing a lung consolidation in the left mid to lower lung zone with an air-fluid level. Computed tomography of the chest with contrast was performed, and he was then started on broad-spectrum antibiotics and admitted with the diagnosis of pulmonary abscess and COVID-19 infection. 

**Figure 2:**
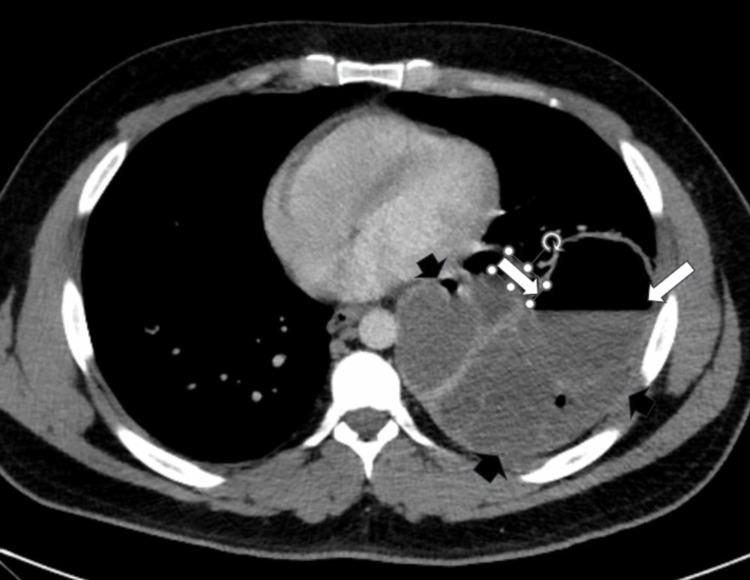
Admission chest CT scan Axial contrast-enhanced CT angiogram shows a large low attenuation lesion, which was measured as fluid density based on Hounsfield units with enhancing septations (short black arrows) with air-fluid level (long white arrows).

**Figure 3:**
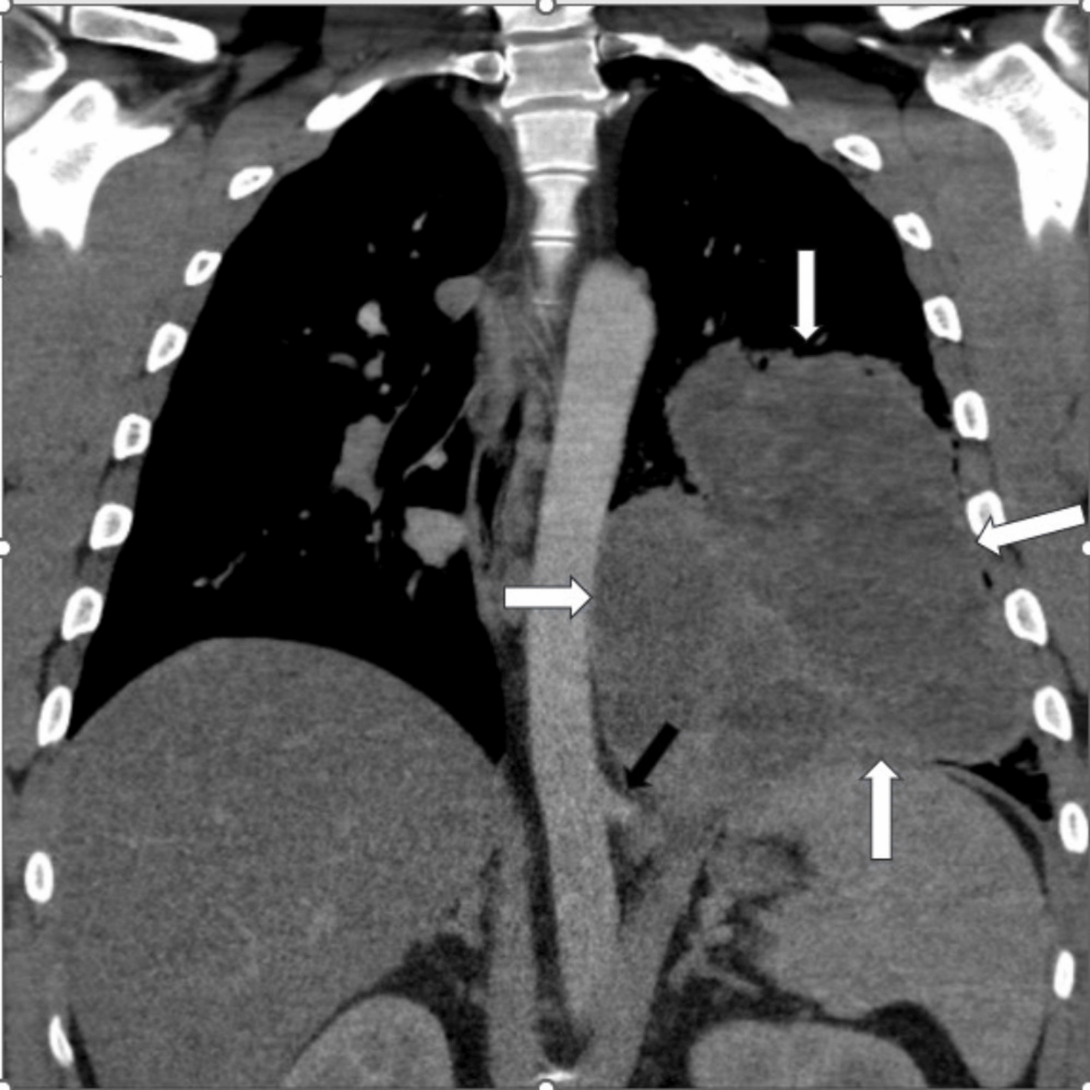
Admission chest CT scan Coronal contrast-enhanced pulmonary CT angiogram shows a large low attenuation lesion measuring fluid density with enhancing septations (long white arrows). There is partial visualization of an aberrant vessel arising from the thoracic aorta (long black arrow).

**Figure 4:**
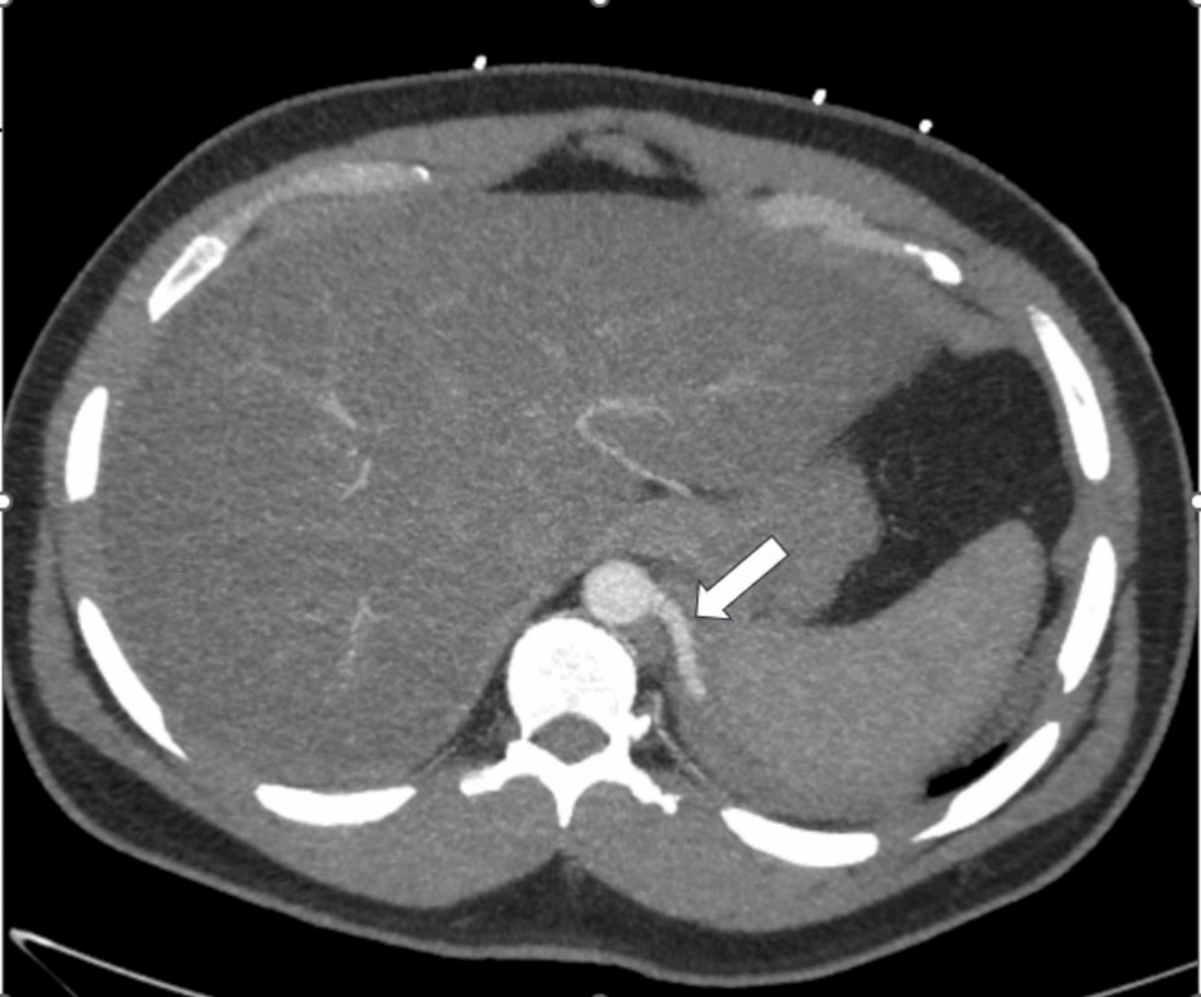
Admission chest CT scan Axial contrast enhanced pulmonary CT angiogram shows an aberrant artery arising from the distal descending thoracic aorta.

**Figure 5:**
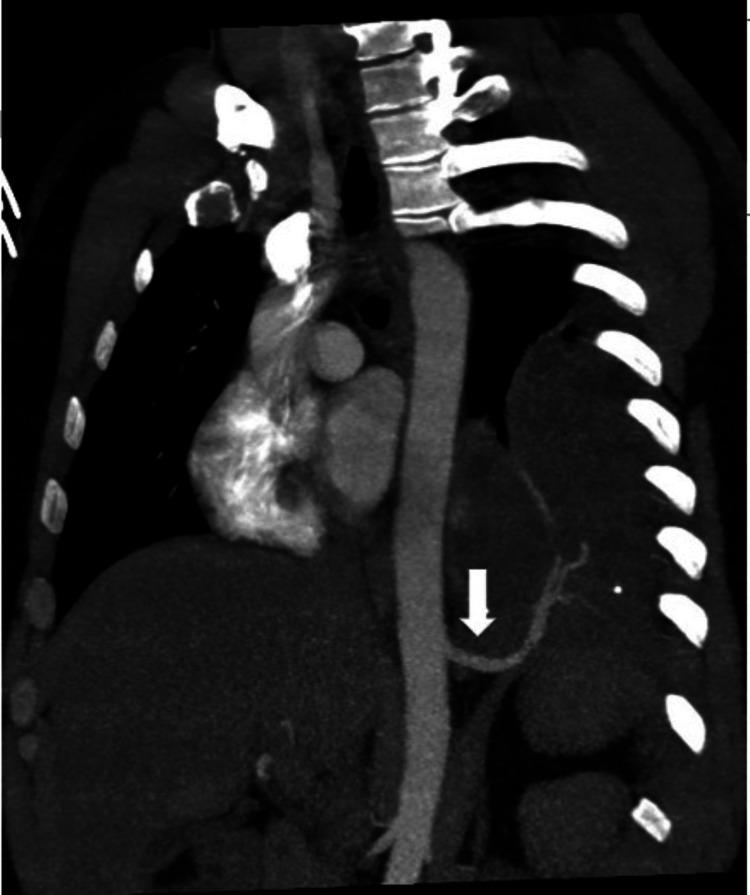
Admission chest CT scan Coronal oblique maximum intensity projection (MIP) reconstructions show an aberrant artery arising from the distal thoracic aorta above the diaphragm. This vessel courses cephalad to supple the sequestration.

Figures 2-5 show the CT images, which revealed a multi-lobulated cystic structure that was receiving arterial blood supply from a branch of the aorta. With these findings, the leading diagnosis changed from abscess to intralobar pulmonary sequestration. Upon repeated questioning, the patient denied receiving this diagnosis in the past. However, we obtained prior medical records from his admission for abdominal surgery and found that his prior abdominal CT scan did note an incidental finding of pulmonary sequestration. 

He was ultimately diagnosed with infected pulmonary sequestration in addition to COVID-19. He was hospitalized for several weeks, treated with IV antibiotics piperacillin/tazobactam 3.375 g IV every six hours, and transitioned to oral antibiotics amoxicillin/clavulanate 875 mg/125 mg PO twice daily and levofloxacin 750 mg PO daily for one to two months once he was well enough to resume his drive. He was referred to a university-based pulmonary clinic in his home state for follow-up.

## Discussion

First described in the 1940s by Dr. Pryce [3], pulmonary sequestration is divided into intra- and extra-lobar types. Intralobar sequestration is located within the visceral pleura, whereas extralobar sequestration resides within its own pleura. Developmentally, pulmonary sequestration is most likely thought to arise from an accessory supernumerary lung bud below the true lung bud that continues to migrate caudally with the true lung bud [4]. If arising before pleural development, it becomes intralobar; otherwise, it develops extralobarly. 

The arterial supply of the accessory bud is anomalous. Case reviews have shown that 74% of intralobar sequestration has arterial supply arising from the thoracic aorta [4]. In extralobar cases, the arterial supply tends to be from branches of the aorta. The venous return in intralobar sequestration may occur through the pulmonary veins but also can occur through the azygos system, portal vein, directly into the right atrium, or via the IVC. Extralobar sequestration most commonly drains into the right atrium through systemic veins.

Extralobar sequestration is more rare and accounts for 15-25% of cases [5]. It usually presents in the neonatal period as respiratory distress, high output cardiac failure (due to R->L shunting), and occasional pulmonary or pleural hemorrhage [6].

Intralobar sequestration is more common, accounting for the remaining 75-86% of cases [7]. It is found most often in the posterior basal segment of the lower lobe of the left lung. Intralobar sequestration tends to present later in life; the average age of diagnosis is 42 years old [8]. Around 30% of radiographically identified cases are incidental findings. Common presenting symptoms are recurrent pneumonia in the abnormal lung segment, but symptoms may also present as persistent cough, back pain, hemoptysis, or exertion dyspnea. Nearly half of adults with intralobar sequestration are asymptomatic.

Ultrasound may pick up on the anatomical abnormalities, even as early as 16-week antenatal ultrasound. CT chest with contrast may have a variety of presentations, appearing as a mass, a cyst, an encapsulated lesion with air-fluid level, focal atelectasis, bronchiectasis, or a lamellar (flat) lesion. Given that 20% of cases may be infra-diaphragmatic, it may be difficult to differentiate from a variety of other diagnoses. Highlighting the diagnostic complexity, a retrospective review including 72 cases by Xiao et al. showed that a diagnosis was not made until surgery in 62% of cases, despite advanced imaging [9]. 

Recommended treatment for established pulmonary sequestration is lobectomy even in asymptomatic patients to avoid infection risk and progressive inflammation [2]. However, evidence is lacking that there is a clear benefit in preemptive surgery vs. a conservative approach for the asymptomatic patient.

Resection may be done by open thoracotomy or video-assisted thoracoscopic surgery (VATS). More recently, endovascular embolization has become another surgical alternative.

Pulmonary sequestration is a rare congenital disorder but one whose imaging findings can easily be misidentified as abscess or even malignancy, despite advanced imaging, a fact that is highlighted by the number of cases in which a final diagnosis was not made until surgical intervention [10].

Careful review of the vascular supply to lesions was key to our patient’s diagnosis. While most cases of sequestration are present in childhood, the presentation in some adult patients, particularly those with intralobar sequestration (as in our case), makes understanding this topic relevant for practitioners across the age continuum.

Given that few patients have other developmental abnormalities and have most commonly been asymptomatic until presentation, a careful family history or pediatric medical history may not provide any clues. Further, close to a third of cases may be reported only as incidental findings, as was the case for our patient on his CT scan done for diverticular disease years ago. This emphasizes the importance of ensuring appropriate follow-up for patients with incidental findings. 

We were unable to find guidance in the literature on the duration of antibiotics for infected pulmonary sequestrations, and his treatment was ultimately similar to that recommended for pulmonary abscess.

## Conclusions

Pulmonary sequestration is a rare congenital anomaly in which extralobar type presents in the neonatal period as high output cardiac failure, respiratory distress, and occasional pulmonary hemorrhage, while intralobar type is usually diagnosed later in life and frequently found as an incidental finding. Radiographic findings can be misleading as abscess or malignancy. Accurate diagnosis becomes more difficult if the patient presents with other infections, such as viral infection in our patient. A careful review of radiographic imaging is very important to identify vascular supply to the lesion in question. Further guidance in the literature on treatment durations for infection, indications for and timing of surgery, and the role of less invasive interventions is needed. 
